# Complement C5a Promotes Epithelial-Mesenchymal Transition in Pterygium via C5aR Activation

**DOI:** 10.1167/iovs.67.6.16

**Published:** 2026-06-10

**Authors:** Jiaxin Han, Qianwen Gong, He Wang, Bintao Xie, Lingying Ye, Lijun Shen, Liang Hu

**Affiliations:** 1 National Clinical Research Center for Ocular Diseases, Eye Hospital, Wenzhou Medical University, Wenzhou, People's Republic of China; 2 State Key Laboratory of Ophthalmology, Optometry, and Visual Science, Eye Hospital, Wenzhou Medical University, Wenzhou, People's Republic of China; 3 Department of Ophthalmology, the Affiliated Hospital of Xuzhou Medical University, Xuzhou, Jiangsu Province, People's Republic of China; 4 Department of Ophthalmology, Zhejiang Provincial People’s Hospital, Zhejiang, People's Republic of China

**Keywords:** pterygium, C5a, C5aR, epithelial-mesenchymal transition (EMT), VEGFA

## Abstract

**Purpose:**

The purpose of this study was to investigate candidate regulatory proteins associated with the pathogenesis of pterygium and to explore their potential biological roles.

**Methods:**

Paired pterygium and conjunctival tissues from the discovery cohort (*n* = 10) and the validation cohort (*n* = 3) were subjected to proteomic analysis (label-free/4D label-free) to identify candidate differentially expressed proteins (DEPs). Exploratory clinical correlation and diagnostic analyses of DEPs were performed. Functional validation was conducted using primary conjunctival epithelial cells (PCECs) treated with C5a with or without the C5aR antagonist PMX53. Assays included evaluation of epithelial-mesenchymal transition (EMT) markers (RT-qPCR, Western blot, and immunofluorescence), migration (wound-healing assay), apoptosis (flow cytometry), and VEGFA expression.

**Results:**

Proteomic analysis showed that C5 was consistently upregulated, and upregulated DEPs were significantly enriched in the complement and coagulation cascades**.** Furthermore, C5 showed a significant positive correlation with vessel density (*r* = 0.68, *P* = 0.029) and achieved an area under the curve (AUC) of 0.8 in the discovery cohort. Functional validation demonstrated that C5a promoted EMT-like marker changes in PCECs, including decreased E-cadherin and increased vimentin and fibronectin (FN). C5a treatment was also associated with enhanced cellular migration and reduced apoptosis, and these cellular changes were attenuated by the C5aR antagonist PMX53. Notably, C5a also increased VEGFA expression.

**Conclusions:**

These findings suggest that C5a/C5aR signaling may contribute to pterygium pathogenesis by promoting EMT-related changes in conjunctival epithelial cells, supporting C5aR as a potential therapeutic target.

Pterygium is a prevalent ocular surface disease characterized by fibrovascular growth of conjunctival tissue onto the cornea, affecting approximately 200 million individuals globally, with an estimated prevalence of 12%.^1^^,^^2^ Beyond cosmetic concerns, it can cause ocular discomfort, restricted eye movement, and even vision loss (14.3% prevalence of visual impairment) or blindness (3.9% prevalence of blindness) due to corneal astigmatism or pupillary occlusion, significantly impairing patients’ quality of life.^3^ Currently, surgical excision remains the primary treatment, but it is associated with high recurrence rates (up to 89%).^4^^,^^5^ Adjuvant drug therapies, although used in combination with surgery, are limited by significant side effects or high costs.^6^^,^^7^ Therefore, elucidating its pathogenesis to guide prevention and recurrence control remains an important clinical challenge.

Current evidence indicates that chronic exposure to environmental insults, such as ultraviolet radiation and dust, may trigger sustained immune-inflammatory responses on the ocular surface, thereby contributing to pterygium development. Within this inflammatory milieu, infiltrating immune cells, such as macrophages and lymphocytes, release abundant inflammatory mediators.^8^ These stimuli collectively drive a series of pathological cellular behaviors in pterygium tissues, including enhanced cell migration, increased cell proliferation, resistance to apoptosis, extracellular matrix (ECM) remodeling, and angiogenesis.^9^^,^^10^ These functional alterations contribute to the fibrovascular proliferation and invasive growth characteristic of pterygium.

Among these mechanisms, epithelial-mesenchymal transition (EMT) has been recognized as a key biological process underlying pterygium progression.^7^^,^^11^^,^^12^ EMT refers to a process in which epithelial cells lose polarity and intercellular junctions while acquiring migratory and mesenchymal-like characteristics.^13^^,^^14^ During EMT, epithelial markers such as E-cadherin are downregulated, whereas mesenchymal markers including vimentin and fibronectin (FN) are upregulated.^14^ Multiple signaling pathways have been implicated in regulating EMT in ocular surface diseases, including TGF-β, Wnt, and NF-κB pathways.^15^ This transformation may contribute to the invasive behavior of pterygium tissue. However, the upstream inflammatory mediators that initiate or modulate EMT-related changes in pterygium remain incompletely understood.

To systematically identify candidate molecular mediators involved in pterygium pathogenesis, this study used an integrated discovery-validation approach beginning with exploratory proteomic screening of paired pterygium and conjunctival tissues. Complementary label-free and 4D label-free proteomic workflows were used to identify differentially expressed proteins (DEPs). Building on these proteomic findings, we focused on C5a, the bioactive fragment of complement component C5, and examined its potential effects on human primary conjunctival epithelial cells (PCECs) through mechanistically oriented assays. This study aimed to explore whether C5a/C5aR signaling is associated with EMT-related epithelial changes in pterygium and to provide evidence supporting potential therapeutic targets for future investigation.

## Methods

### Study Population

This prospective study enrolled 13 consecutive patients with primary nasal pterygium at the Eye Hospital of Wenzhou Medical University between July 2022 and March 2023. The diagnosis was established by slit-lamp examination performed by an experienced ophthalmologist (author L.L.Y.) based on the typical clinical features of a triangular fibrovascular tissue originating from the bulbar conjunctiva and extending onto the cornea. Inclusion criteria were: (1) primary nasal pterygium with corneal extension greater than 1 mm; (2) no history of pterygium surgery; and (3) presence of a clinical indication for surgical excision. Exclusion criteria were strictly applied, including previous ocular surgery, recurrent pterygium, ocular trauma, contact lens use (≤3 months), active conjunctivitis, corneal scarring, glaucoma, and systemic disorders (e.g., hypertension and diabetes). The study protocol was approved by the Institutional Review Board of Wenzhou Medical University (No. 2022-126-K-96-01) and conformed to the Declaration of Helsinki.

### Clinical Data Collection

Preoperatively, slit-lamp microscopy examination was performed to assess the presence of ocular diseases and measure pterygium length, area, and thickness. Optical coherence tomography angiography (OCTA; VG100D; SVision Imaging) was used to measure pterygium vessel density. Functional slit-lamp biomicroscopy (FSLB) was used to measure the following vascular parameters: vessel diameter (µm), vessel length (µm), axial blood flow velocity (Va, mm/s), cross-sectional blood flow velocity (Vs, mm/s), and blood flow volume (Q, pl/s). All imaging acquisition and quantitative analyses were performed according to the standardized protocols described in our previous study.^16^

### Sample Collection

Pterygium and conjunctiva tissues were collected intraoperatively by the same surgeon (author L.L.Y.). Samples were immediately stored in sterile Eppendorf tubes at −80°C until analysis. Normal bulbar conjunctival tissues were obtained from an eye bank, specifically from the bulbar conjunctiva of 14 eyes from 7 donors. When sufficient tissue was available, conjunctival tissue from the same eye was subdivided so that separate portions could be used for different experiments, including PCEC culture and paraffin embedding. Informed consent was obtained from the families of all donors prior to tissue collection. The human tissue experiments complied with the guidelines of the ARVO Best Practices for Using Human Eye Tissue in Research (November 2021). All tissue procurement procedures complied with ethical review requirements, and written informed consent was obtained from all patients and donors’ families.

### Proteomic Analysis

**Figure 1.**
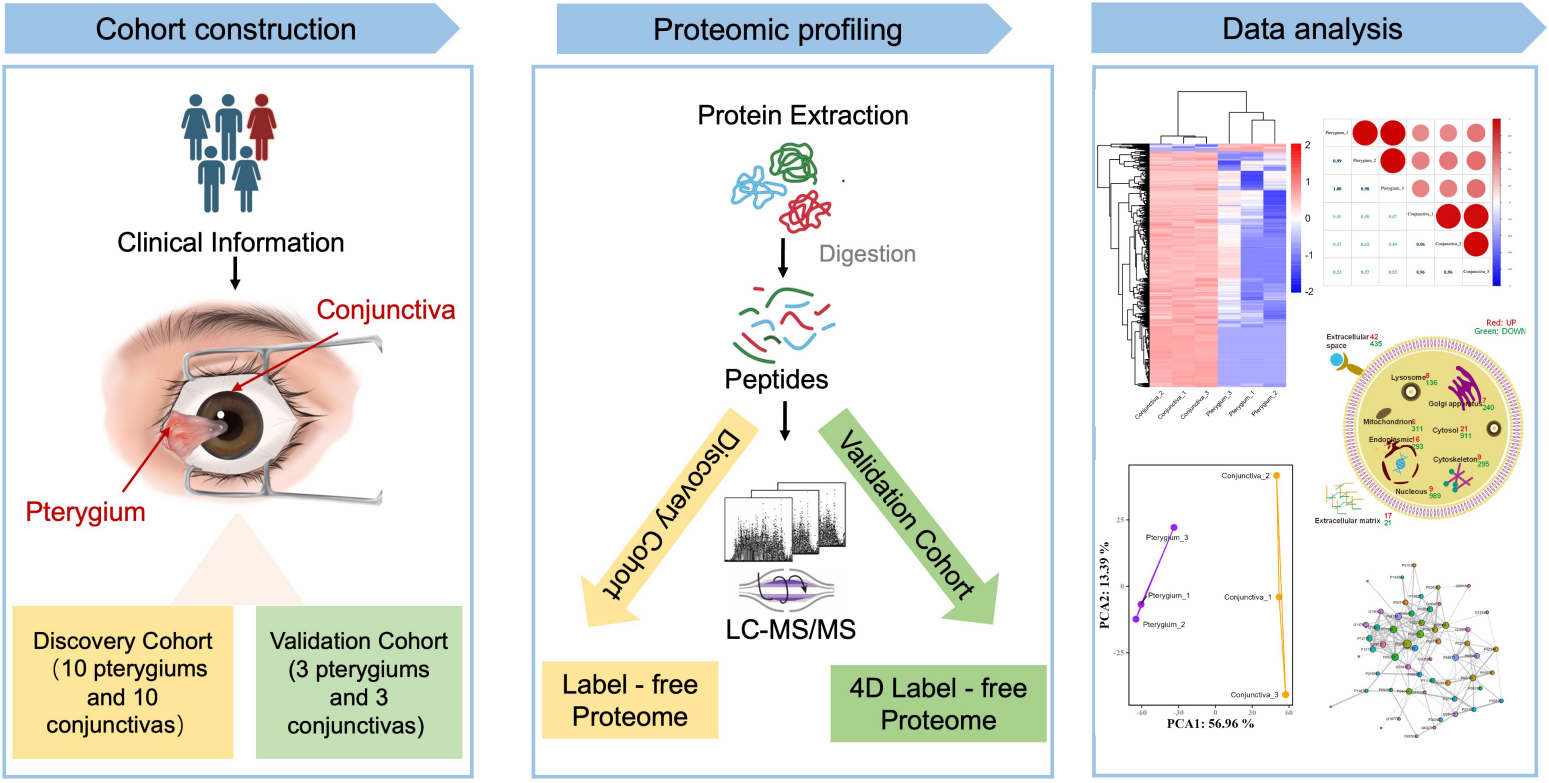
**Schematic of the proteomic analysis workflow in pterygium research.** The study used a dual-cohort design with a discovery cohort (10 paired pterygium and normal conjunctival tissues) and an independent validation cohort (*n* = 3). Proteins were extracted, enzymatically digested into peptides, and analyzed by liquid chromatography-tandem mass spectrometry (LC-MS/MS) using label-free proteomics (discovery cohort) and 4D label-free proteomics (validation cohort). Following quality control, differential expression profiling of pterygium versus conjunctival proteomes was performed to ensure data reliability.

This study used a two-phase proteomic strategy. The discovery cohort included 10 matched pterygium and normal conjunctival tissue pairs and was used for broad proteomic screening; therefore, a relatively larger sample size was adopted to improve the stability of differential protein identification. The validation cohort consisted of three additional matched tissue pairs and was analyzed independently using a different and more sensitive 4D label-free proteomic platform. This cohort was designed to assess whether the key findings from the discovery stage could be reproduced in an independent sample set and across analytical platforms. The integrated experimental design is schematically presented in Figure 1. Tissue proteins were digested into peptides for liquid chromatography-tandem mass spectrometry (LC-MS/MS) analysis. For the discovery cohort, label-free proteomic analysis was performed at the State Key Laboratory of Wenzhou Medical University using a mass spectrometer (Orbitrap Fusion Lumos Tribrid; Thermo Fisher Scientific). For the validation cohort, 4D label-free proteomic analysis was performed independently at Shanghai Aipudikang Laboratory, using a mass spectrometer (Orbitrap Exploris 480; Thermo Fisher Scientific) with High-field Asymmetric Waveform Ion Mobility Spectrometry (FAIMS). Detailed LC-MS/MS acquisition parameters for both cohorts, including the LC system, column configuration, gradient conditions, flow rate, scan settings, resolution, and FAIMS compensation voltages, are provided in Supplementary File S1. Raw data were processed with Proteome Discoverer against the UniProt database. Missing values were imputed with one-tenth of the minimum value across the dataset. Student's *t*-test was used to identify significant differences. Proteins with *P* < 0.05 and fold change (FC) >2 or <0.5 were defined as DEPs. Functional enrichment of DEPs was analyzed via Gene Ontology (GO) and Kyoto Encyclopedia of Genes and Genomes (KEGG) pathways.

### Enzyme-Linked Immunosorbent Assay 

Tissue samples from pterygium and conjunctiva were collected to quantify the levels of complement C5 and C5a. ELISA was performed using commercially available kits (Wuhan Mosak Biotech, Cat. # KT99032 and KT40003) according to the manufacturer's instructions. Each sample was processed and analyzed individually.

### Hematoxylin and Eosin Staining and Immunohistochemical Staining

Tissue samples from pterygium and normal conjunctiva were fixed in 4% paraformaldehyde, dehydrated, paraffin-embedded, and sectioned at 5-µm thickness. For hematoxylin and eosin (H&E) staining, sections were processed using a Leica Autostainer XL (ST5010; Leica Microsystems).

For immunohistochemistry (IHC), after dewaxing, rehydration, and antigen retrieval in sodium citrate buffer (95°C, 15 minutes), endogenous peroxidase was blocked with 3% hydrogen peroxide. Sections were then incubated with 5% bovine serum albumin (BSA) and subsequently with primary antibodies against complement C5a receptor 1 (C5aR1, hereafter referred to as C5aR; 1:100) or vascular endothelial growth factor A (VEGFA; 1:100) overnight at 4°C. After phosphate-buffered saline (PBS) washes, immunodetection was performed using the Elivision plus Polyer HRP IHC Kit (Maixin Biotech, Cat. #KIT-9901) with DAB chromogen (Maixin Biotech, Cat. #DAB-0031), strictly according to the manufacturer's instructions. Detailed antibody and reagent information are listed in Table 1. IHC was semi-quantitatively analyzed in ImageJ software (NIH, Bethesda, MD, USA) using a threshold-based DAB method.^17^ The tissue area of interest was manually selected as the region of interest (ROI), excluding empty background and tissue folds. Integrated density (IntDen) and area were measured after DAB thresholding, and average optical density (AOD) was calculated as IntDen/area. Five representative non-overlapping fields were analyzed for each sample, and the mean value was used for statistical analysis.

**Table 1.** Recombinant Proteins and Primary Antibodies Used in the Study

Product Name	Cat. No	Company
Recombinants/molecules		
Recombinant human complement C5a protein	2037-C5-025	R&D Systems
PMX 53 (C5a receptor antagonist)	S6239	Selleck chemicals
Antibodies		
Pan-keratin	26411-1-AP	Proteintech
C5aR	ab252435	Abcam
VEGFA	ab46154	Abcam
GAPDH	60004-1-Ig	Proteintech
E-cadherin	3195	Cell signaling
Vimentin	10366-1-AP	Proteintech
Fibronectin	15613-1-AP	Proteintech

### Cell Culture and Identification

#### Primary Culture of Conjunctival Epithelial Cells

PCECs were derived from healthy bulbar conjunctival tissues obtained from five donors using the explant culture method. Bulbar conjunctiva tissue was rinsed with PBS containing 1% penicillin/streptomycin (Gibco, USA). The epithelial layer was separated from the stromal layer under a microscope. The epithelial tissue was minced into 2 × 2 mm pieces using ophthalmic corneal scissors. The tissue explants were evenly seeded into a six-well plate. The culture plate was placed in a 37°C, 5% CO₂ incubator for 10 to 20 minutes to facilitate attachment of the explants. Subsequently, 2 mL of Dulbecco's modified Eagle's medium/F12 (DMEM/F12; Gibco, USA) culture medium supplemented with 10% fetal bovine serum (FBS; Gibco, USA) and 1% penicillin/streptomycin was added to each well. PCECs at passages 3 to 6 were used for subsequent experiments. For treatment experiments, cells were seeded and allowed to adhere for 24 hours, after which the medium was replaced with low-serum DMEM/F12 containing 1% FBS. After an additional 24 hours, different concentrations of C5a or PMX53 (a pharmacologic C5aR antagonist) were added for the indicated durations.

#### Immunofluorescence for Cell Identification

Immunofluorescence (IF) staining was performed as previously described^18^ with modifications. Briefly, cells seeded on coverslips were fixed with 4% paraformaldehyde, blocked with 5% BSA, and incubated overnight at 4°C with primary antibodies against pan-keratin (1:200) or C5aR (1:50). After PBS washes, cells were incubated with secondary antibodies (1:400) and counterstained with 4,6-diamidino-2-phenylindole (DAPI). Images were acquired using a fluorescence microscope (DM4B; Leica Microsystems).

### Cell Proliferation Assay

PCECs were seeded in 96-well plates and cultured in 1% FBS medium containing varying concentrations (0, 5, 10, 25, 50, 100, and 200 ng/mL) of C5a. Cells were exposed to C5a for different durations (24, 48, 72, and 96 hours), with culture medium refreshed daily. Cell viability was assessed using the Cell Counting Kit-8 (CCK-8; Beyotime Biotechnology, Cat. #C0038). Briefly, 10 µL of CCK-8 solution was added to each well followed by incubation at 37°C for 2 hours. Absorbance was measured at 450 nm using a microplate reader (SpectraMax 190; Molecular Devices).

### Reverse Transcription Quantitative Polymerase Chain Reaction

Total RNA was extracted using the FastPure Cell/Tissue Total RNA Isolation Kit (Vazyme Biotech, Cat. #RC112-01) according to the manufacturer's instructions. RNA was reverse-transcribed into cDNA using the HiScript III RT SuperMix kit (Vazyme Biotech, Cat. #R323-01). Reverse transcription quantitative polymerase chain reaction (RT-qPCR) was performed on cDNA using ChamQ Universal SYBR qPCR Master Mix (Vazyme Biotech, Cat. #Q711-03) on a real-time PCR system (Q6; Applied Biosystems). The relative transcription was analyzed using the 2^−ΔΔCt^ method with β-actin as an internal reference gene. Primer sequences are listed in Table 2.

**Table 2.** RT-qPCR Primer Sequences

Gene Name	Forward (5′-3′)	Reverse (5′-3′)
*Vimentin*	CTTGACGGAAGACATAGTACACC	ACATTGGCAGGAAATAGTCGC
*Fibronectin*	CGGTGGCTGTCAGTCAAAG	AAACCTCGGCTTCCTCCATAA
*E-cadherin*	AAAGGCCCATTTCCTAAAAACCT	TGCGTTCTCTATCCAGAGGCT
*VEGFA*	AGGGCAGAATCATCACGAAGT	AGGGTCTCGATTGGATGGCA
*β-actin*	CATGTACGTTGCTATCCAGGC	CTCCTTAATGTCACGCACGAT

### Western Blotting and Immunofluorescence

For Western blotting (WB) and IF analyses, PCECs were treated for 96 hours with daily medium replacement under 3 experimental conditions: (1) 50 ng/mL C5a in DMEM/F12, (2) DMEM/F12 vehicle control, and (3) 50 ng/mL C5a plus 50 nM PMX53 in DMEM/F12. WB was performed according to established methodology using primary antibodies against GAPDH (1:10000), E-cadherin (1:1000), vimentin (1:1000), and FN (1:1000).^18^ IF procedures used antibodies targeting E-cadherin (1:1600), vimentin (1:400), and FN (1:400), with complete antibody sourcing provided in Table 1.

### Wound Healing Assay

PCECs were cultured to full confluence, followed by 24-hour serum reduction. A standardized wound was created in the monolayer using a sterile 200-µL pipette tip. Following 3 PBS washes, the cells were treated identically to section 10 groups. The wound was photographed immediately and 12 hours after the scratch. Images were analyzed using ImageJ software.

### Annexin V-FITC/PI Apoptosis Detection

PCECs were assessed for apoptosis using an Annexin V-FITC/propidium iodide (PI) detection kit (Vazyme Biotech, Cat. #A211) following the manufacturer’s protocols. Cells were dually stained with Annexin V-FITC and PI, then analyzed within 1 hour using a flow cytometer (BD Accuri C6 Plus; BD Biosciences). Apoptotic populations were defined as: Annexin V-FITC⁺/PI⁻ (early apoptosis) and Annexin V-FITC⁺/PI⁺ (late apoptosis).

### Statistical Analysis

Statistical analyses were performed using SPSS 29.0 (IBM Corp., Armonk, NY, USA). Spearman's rank-order correlation (2-tailed) was performed using the discovery cohort dataset to assess relationships between protein expression levels and clinical characteristics. Receiver operating characteristic (ROC) curve analysis was also performed using the discovery cohort dataset to evaluate the diagnostic performance of upregulated proteins, and the area under the curve (AUC) with 95% confidence intervals (CIs) was calculated.

Semi-quantitative immunohistochemical data, ELISA data, and in vitro experimental data were analyzed using GraphPad Prism 8 (San Diego, CA, USA). Comparisons between two groups were performed using an unpaired two-tailed t-test, whereas comparisons among three groups were performed using 1-way ANOVA followed by Tukey's multiple comparisons test. All experiments included greater than or equal to three independent biological replicates. Continuous data are expressed as mean ± standard deviation (SD). Statistical significance was defined as P < 0.05.

## Results

### Patient Characteristics and Clinical Features

Thirteen patients were enrolled in this study, including 10 in the discovery cohort for label-free proteomic analysis and 3 in the independent validation cohort for 4D label-free proteomic profiling. The baseline demographic and clinical characteristics of the two cohorts are summarized in Table 3. Comparative analysis showed no significant differences between the discovery and validation cohorts in sex, age, disease duration, pterygium length, area, thickness, vessel density, vessel diameter, vessel length, Va, Vs, or Q (all *P* > 0.05).

**Table 3.** Baseline Demographic and Clinical Characteristics of the Discovery and Validation Cohorts

Variable	Discovery Cohort (*n* = 10)	Validation Cohort (*n* = 3)	Test Statistic	*P* Value
Sex, M/F	6/4	2/1	—	1.000
Age, y	61.40 ± 12.20	62.00 ± 10.58	*t* = −0.076	0.940
Disease duration, y	7.60 ± 3.20	7.00 ± 2.65	*U* = 14.000	0.937
Pterygium length, mm	3.90 ± 1.20	3.87 ± 1.67	t = 0.039	0.970
Pterygium area, mm²	17.65 ± 8.83	17.64 ± 12.11	t = 0.002	0.999
Pterygium thickness, µm	433.25 ± 117.49	456.41 ± 44.39	U = 15.000	1.000
Vessel density, %	69.39 ± 5.05	71.01 ± 1.78	t = −0.531	0.606
Vessel diameter, µm	16.89 ± 1.17	15.76 ± 1.62	t = 1.360	0.201
Vessel length, µm	162.30 ± 16.57	142.10 ± 15.33	U = 5.000	0.112
Va, mm/s	0.65 ± 0.28	0.57 ± 0.09	t = 0.523	0.611
Vs, mm/s	0.46 ± 0.19	0.41 ± 0.06	t = 0.413	0.688
Q, pl/s	146.12 ± 68.21	84.56 ± 34.72	t = 1.474	0.169

Data are presented as mean ± SD unless otherwise indicated. Continuous variables were compared using independent-samples *t*-tests, except for disease duration, pterygium thickness, and vessel length, which were analyzed using Mann-Whitney *U* tests. Sex distribution was compared using Fisher's exact test.

### Proteomic Results

#### Protein Identification and Screening of DEPs

**Figure 2.**
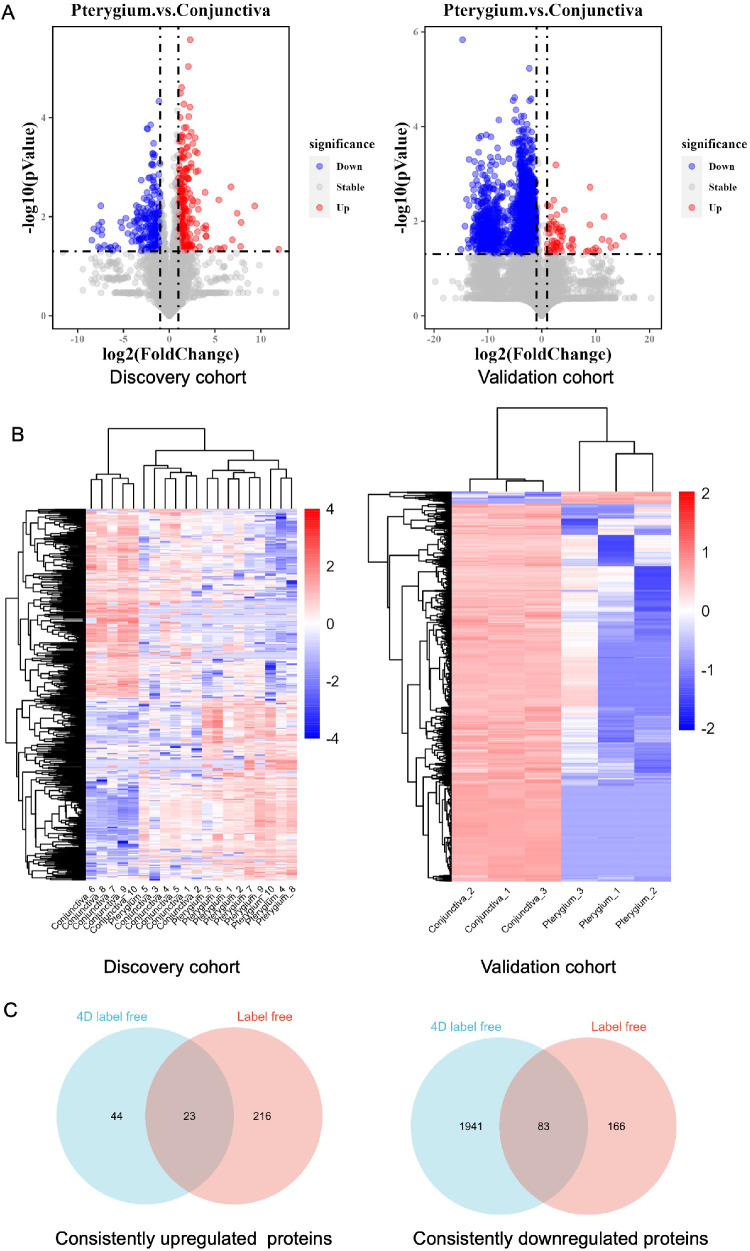
**Proteomic profiling of pterygium versus normal conjunctiva across discovery and validation cohorts.** (**A**) Volcano plots of differentially expressed proteins (DEPs). The discovery cohort (*left*) identified 239 upregulated (*red*) and 249 downregulated (*blue*) proteins, whereas the validation cohort (*right*) identified 67 upregulated and 2024 downregulated proteins. (**B**) Hierarchical clustering heatmaps of DEPs showing distinct expression patterns between pterygium and conjunctival tissues in the discovery cohort (*left*) and validation cohort (*right*). (**C**) Cross-platform comparison identified 23 consistently upregulated proteins (*left*) and 83 consistently downregulated proteins (*right*) shared between the two proteomic workflows.

The discovery cohort (label-free proteomics, *n* = 10) identified 4080 proteins, whereas the validation cohort (4D label-free proteomics, *n* = 3) detected 7258 proteins. Differential expression analysis revealed 239 upregulated and 249 downregulated proteins in pterygium versus conjunctiva within the discovery cohort, contrasted with 67 upregulated and 2024 downregulated proteins in the validation cohort (Fig. 2A). Hierarchical clustering of DEPs demonstrated high intra-group homogeneity and distinct inter-group separation (Fig. 2B). Cross-platform comparison identified 23 consistently upregulated proteins (Table 4) and 83 consistently downregulated proteins (Fig. 2C), highlighting candidate molecules potentially involved in pterygium pathogenesis.

**Table 4.** The 23 Consistently Upregulated Proteins

Gene ID	Gene Name	Gene Description	FC-1	FC-2	*P*-1	*P*-2
P12111	*COL6A3*	Collagen alpha-3(VI) chain	2.07	2.48	<0.01	0.01
P69905	*HBA1; HBA2*	Hemoglobin subunit alpha	2.20	8.02	0.01	0.02
P02549	*SPTA1*	Spectrin alpha chain, erythrocytic 1	2.44	47.88	0.01	0.03
Q99715	*COL12A1*	Collagen alpha-1 (XII) chain	2.12	2.82	<0.01	0.02
P68871	*HBB*	Hemoglobin subunit beta	2.16	14.49	0.01	0.02
P04040	*CAT*	Catalase	2.02	9.65	0.01	0.01
P30043	*BLVRB*	Flavin reductase (NADPH)	2.21	4.53	<0.01	0.01
P02730	*SLC4A1*	Band 3 anion transport protein	2.45	12.47	<0.01	0.03
P00915	*CA1*	Carbonic anhydrase 1	2.76	15.51	0.01	0.02
P01031	*C5*	Complement C5	2.11	2.98	0.01	<0.01
P00918	*CA2*	Carbonic anhydrase 2	2.37	18.48	0.01	0.01
P09493	*TPM1*	Tropomyosin alpha-1 chain	2.27	2.85	<0.01	0.05
P49247	*RPIA*	Ribose-5-phosphate isomerase	2.64	2.07	<0.01	0.03
P14555	*PLA2G2A*	Phospholipase A2, membrane associated	7.10	2.09	<0.01	0.05
Q9UGT4	*SUSD2*	Sushi domain-containing protein 2	2.36	6.19	0.03	0.05
P02100	*HBE1*	Hemoglobin subunit epsilon	3.98	6.46	<0.01	0.04
Q16775	*HAGH*	Hydroxyacylglutathione hydrolase, mitochondrial	2.02	2.06	<0.01	0.01
Q63ZY3	*KANK2*	KN motif and ankyrin repeat domain-containing protein 2	3.67	4.98	0.02	0.04
P35612	*ADD2*	Beta-adducin	3.13	51.60	<0.01	0.02
Q08495	*DMTN*	Dematin	3.39	53.10	<0.01	0.05
O14495	*PLPP3*	Phospholipid phosphatase 3	3.90	2.09	0.03	<0.01
P07996	*THBS1*	Thrombospondin-1	2.06	58.07	0.01	0.03
P00740	*F9*	Coagulation factor IX	2.09	40.42	0.01	0.04

FC-1 = fold change value calculated from label-free proteomics (discovery cohort); FC-2 = fold change value calculated from 4D-label free proteomics (validation cohort); *P*-1 = *P* value derived from label-free proteomics analysis (discovery cohort); *P*-2 = *P* value derived from 4D-label free proteomic analysis (validation cohort).

#### GO Enrichment Analysis of Differentially Expressed Proteins

To systematically explore the biological functions of DEPs between pterygium and conjunctival tissues, we performed GO enrichment analysis in the discovery and validation cohorts, respectively. The enrichment results were then compared across the two cohorts to identify shared biological themes.

##### GO Enrichment Characteristics of Upregulated DEPs

**Figure 3.**
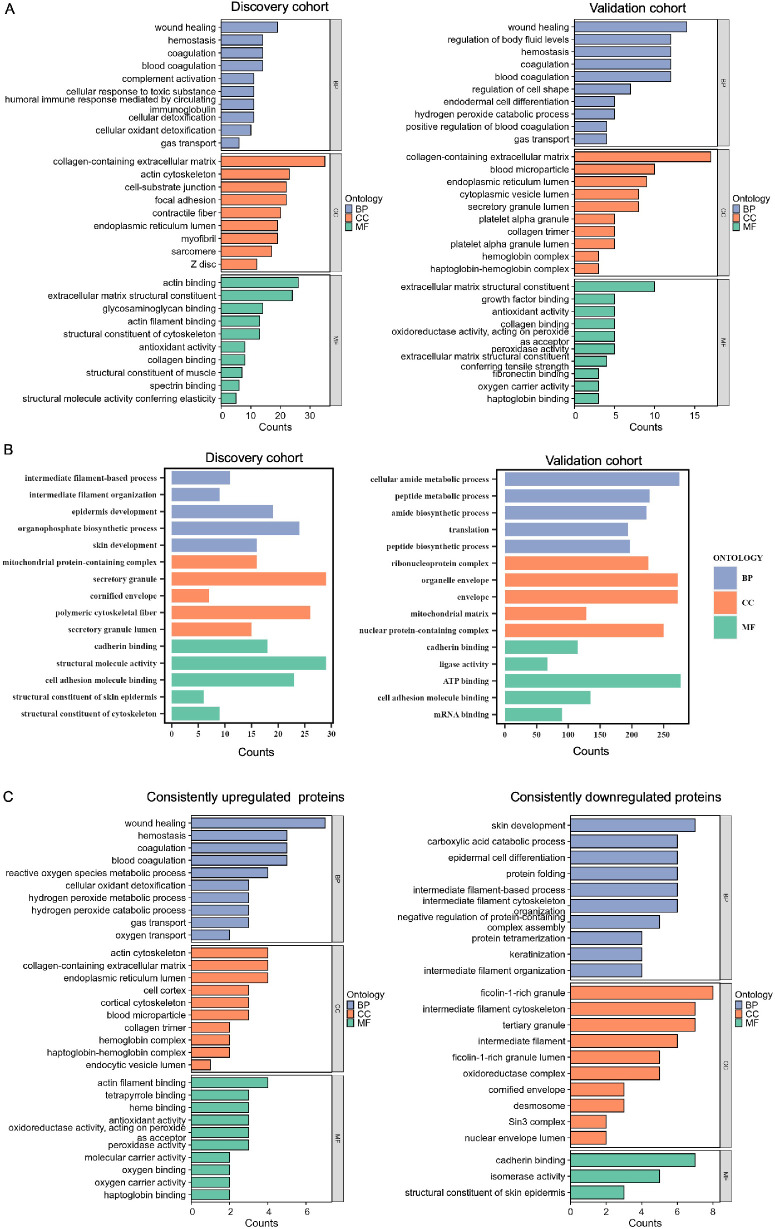
**GO enrichment analysis of differentially expressed proteins (DEPs) in pterygium.** (**A**) GO terms enriched by upregulated DEPs in the discovery cohort (*left*) and validation cohort (*right*). (**B**) GO terms enriched by downregulated DEPs in the discovery cohort (*left*) and validation cohort (*right*). (**C**) Consistently enriched GO terms by shared upregulated DEPs (*left*) and shared downregulated DEPs (*right*) across both cohorts.

As shown in Figure 3A, the GO enrichment results of upregulated DEPs in the discovery and validation cohorts were broadly consistent, confirming the reliability of the data. At the biological process (BP) level, upregulated DEPs were significantly enriched in processes such as hemostasis, coagulation cascades, and gas transport, suggesting blood- and vascular-related biological changes in pterygium tissue. Notably, the discovery cohort identified significant upregulation of immune-related biological processes, particularly complement activation and immunoglobulin-mediated humoral immunity, with core DEPs including complement components C5, C6, C7, C1S, and complement factor D. Among these, C5 was stably upregulated in both the discovery cohort (FC = 2.11, *P* = 0.01) and validation cohort (FC = 2.98, *P* < 0.01), suggesting that it may represent a biologically relevant candidate molecule associated with the immune-inflammatory response in pterygium.

At the cellular component (CC) level, both cohorts consistently showed that upregulated DEPs were enriched in the collagen-containing ECM, involving core molecules such as collagen type VI alpha 3 chain (COL6A3), FN, and MMP-2, supporting the presence of ECM remodeling in pterygium tissues. Further molecular function (MF) analysis revealed that upregulated DEPs were significantly enriched in antioxidant activity, ECM structural constituents, and collagen binding.

##### GO Enrichment Characteristics of Downregulated DEPs

As shown in Figure 3B, downregulated DEPs from both cohorts suggested biological changes related to loss of epithelial characteristics and altered cellular metabolism in pterygium. At the BP level, downregulated DEPs in the discovery cohort were significantly enriched in intermediate filament (e.g., intermediate filament organization and intermediate filament-based processes) and epithelial development-related processes (e.g., epidermal development and skin development), suggesting impaired structural integrity of conjunctival epithelium. The validation cohort further showed that downregulated DEPs were involved in amide metabolism and peptide metabolic pathways, implying abnormal metabolic function in cells. At the CC level, downregulated DEPs were primarily localized to mitochondrial complexes and organelle inner membranes, which are closely related to metabolic functions. At the MF level, one prominent feature was reduced cadherin binding, which may reflect impaired epithelial adhesion and is compatible with epithelial phenotypic alteration.

##### GO Enrichment Validation of Co-Expressed DEPs

As shown in Figure 3C, co-expressed upregulated and downregulated DEPs further supported the shared biological themes observed in both cohorts. Co-upregulated DEPs were enriched in wound healing, hemostasis, coagulation, gas transport, and the collagen-containing ECM, whereas co-downregulated DEPs were enriched in intermediate filament disorganization and loss of cadherin binding. Overall, these findings support coordinated changes related to immune activation, ECM remodeling, and epithelial alteration in pterygium.

#### KEGG Pathway Analysis of Differentially Expressed Proteins

**Figure 4.**
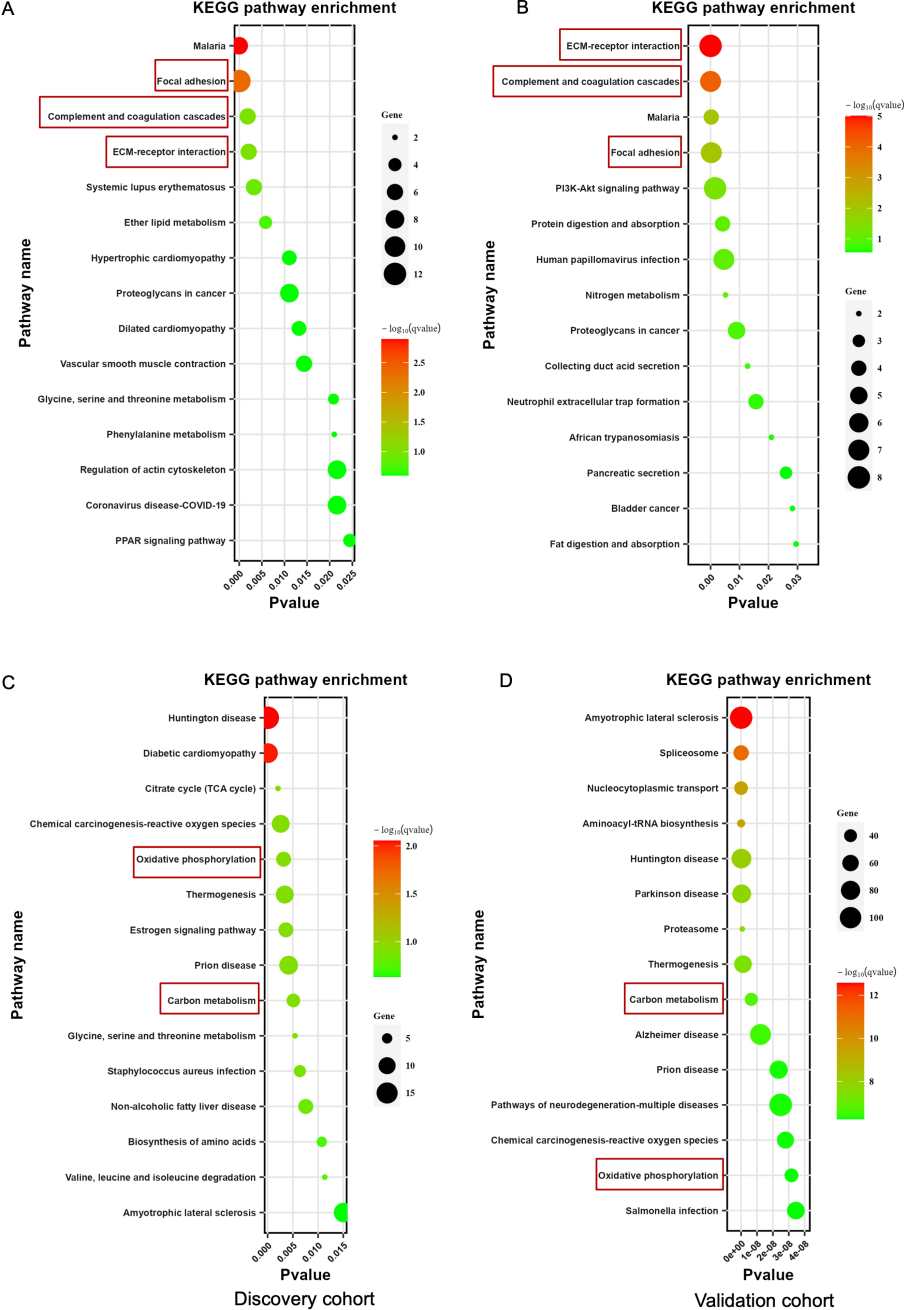
**KEGG pathway enrichment analysis of** **DEPs.** (**A**) Significantly enriched pathways for upregulated proteins in the discovery cohort. (**B**) Enriched pathways for upregulated proteins in the validation cohort. (**C**) Significantly enriched pathways for downregulated proteins in the discovery cohort. (**D**) Enriched pathways for downregulated proteins in the validation cohort.

KEGG enrichment analysis further identified pathways associated with the DEPs. Upregulated DEPs from both the discovery and validation cohorts were enriched in complement and coagulation cascades, ECM-receptor interaction, and focal adhesion pathways (all *P* < 0.01; Figs. 4A, 4B). These pathway-level findings were broadly consistent with the GO enrichment results and support the involvement of complement-related and ECM-associated biological processes in pterygium. Downregulated DEPs were significantly enriched in oxidative phosphorylation, carbon metabolism, and other pathways (Figs. 4C, 4D). Taken together, these enrichment analyses support the involvement of complement activation and ECM-related processes in pterygium and suggest that C5 may be a candidate molecule associated with these biological changes.

### Clinical Relevance and Experimental Validation of C5 and C5a

**Figure 5.**
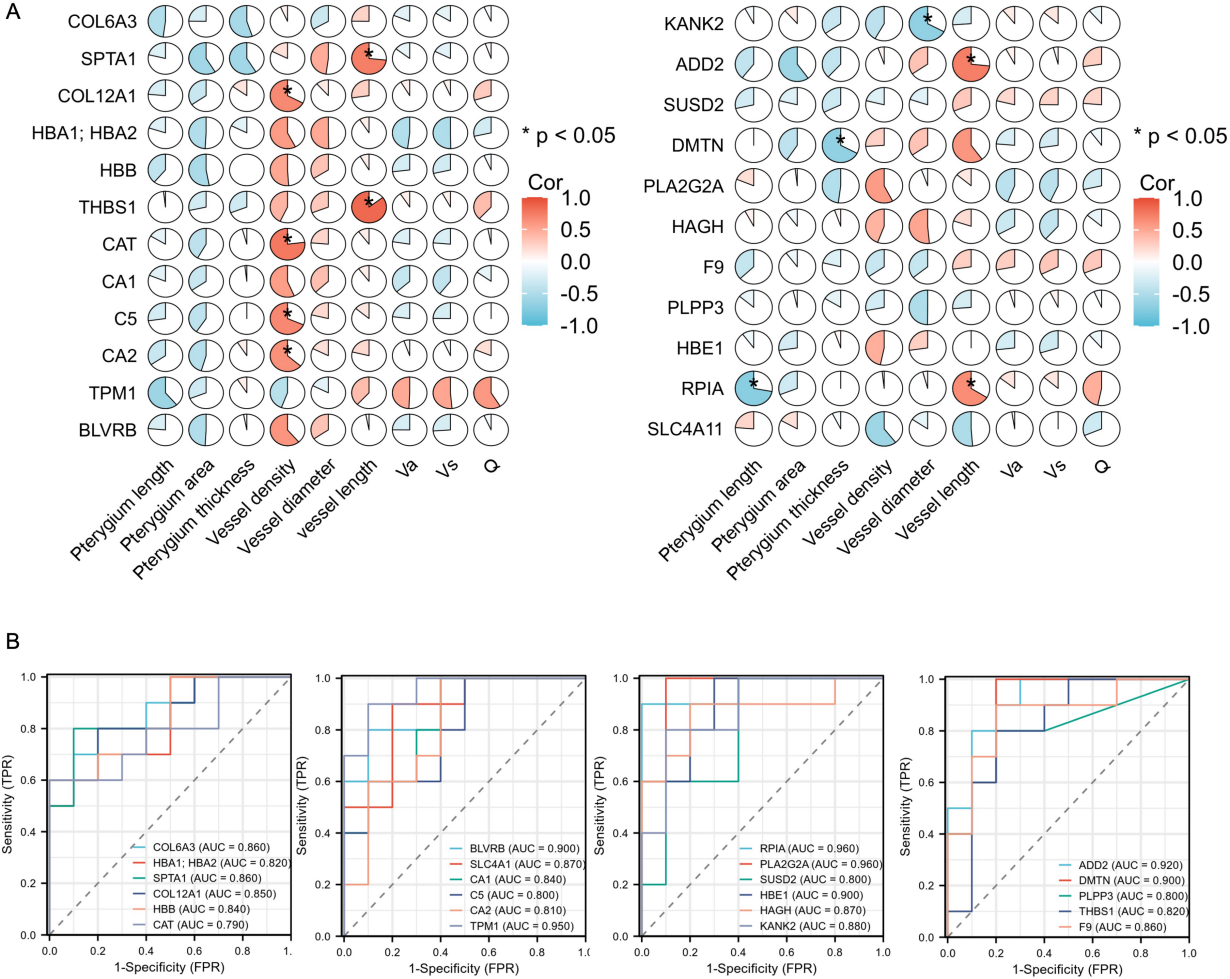
**Correlation and diagnostic value analysis of differentially expressed proteins (DEPs) in pterygium.** (**A**) Correlation heatmap between the 23 consistently upregulated DEPs and clinical parameters of pterygium. Color scale represents Spearman correlation coefficients (Cor), ranging from −1.0 (*blue*, negative correlation) to +1.0 (*red*, positive correlation). Va, axial blood flow velocity; Vs, cross-sectional blood flow velocity; Q, blood flow volume. (**B**) Receiver operating characteristic (ROC) curve analysis of the 23 upregulated DEPs. AUC, area under the curve.

To further evaluate the clinical relevance of the 23 consistently upregulated DEPs, correlation analysis was performed using the discovery cohort (Fig. 5A). Ten DEPs were significantly correlated with one or more clinical parameters (*P* < 0.05), with the most prominent correlations observed with vessel density and vessel length. Thrombospondin-1 (THBS1) showed the strongest positive correlation with vessel length (*r* = 0.85, *P* = 0.002). In addition, four DEPs were significantly positively correlated with vessel density, among which C5 exhibited a relatively strong correlation (*r* = 0.68, *P* = 0.029).

ROC curve analysis was then performed to assess the diagnostic value of the 23 consistently upregulated DEPs. Except for catalase (AUC = 0.79), all DEPs showed AUC values ≥0.80, including C5 (AUC = 0.80), indicating potential discriminatory performance for distinguishing pterygium tissue from normal conjunctiva (Fig. 5B; Supplementary File S2).

Because C5 emerged as a clinically relevant candidate, we next performed ELISA to validate the tissue expression of C5 and its activated fragment C5a. The C5 levels were significantly higher in pterygium tissues than in normal conjunctival tissues (184.29 ± 36.16 µg/mL vs. 70.35 ± 49.57 µg/mL, *P* < 0.01, *n* = 4), supporting the proteomic findings. In addition, C5a concentrations were also significantly elevated in pterygium tissues compared with normal conjunctival tissues (2132.28 ± 246.61 pg/mL vs. 1352.24 ± 192.78 pg/mL, *P* < 0.001, *n* = 6 per group), further supporting increased complement C5 activation in pterygium.

### Histopathological Features and C5aR Expression in Conjunctival Epithelial Cells

**Figure 6.**
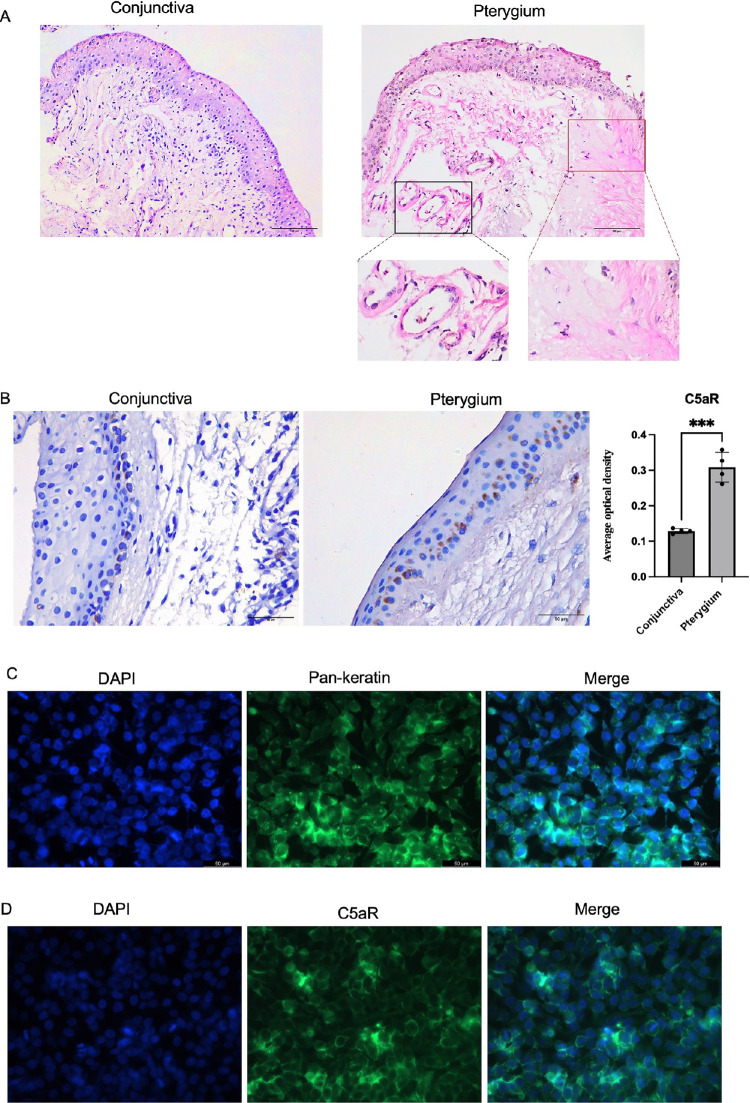
**Cell characterization and tissue histopathology.** (**A**) H&E staining of normal conjunctiva and pterygium. Pterygium stroma (*right*) exhibits collagen degeneration (*red box*), neovascularization (*black box*), and inflammatory infiltrates. (**B**) C5aR IHC in normal conjunctiva and pterygium, with semi-quantitative analysis by average optical density. (**C**) Pan-keratin immunofluorescence in cultured primary conjunctival epithelial cells (PCECs), confirming epithelial identity. (**D**) C5aR immunofluorescence in cultured PCECs, showing membrane-associated expression.

Histopathological alterations in pterygium were characterized using H&E staining. Normal conjunctiva exhibited well-organized epithelial layers overlying a loose connective tissue stroma (Fig. 6A, left). In contrast, pterygium exhibited blurred epithelial cell boundaries, whereas its stromal layer displayed distinct pathological changes: collagen degeneration, diffuse fibroblastic hyperplasia, extensive neovascularization, and inflammatory cell infiltration (see Fig. 6A, right). These pathological features are consistent with the proteomic signatures of ECM remodeling, immune activation, and vascular remodeling. In addition, IHC demonstrated increased C5aR expression in the pterygium epithelium compared with normal conjunctiva, with predominant basal epithelial localization (Fig. 6B). Semi-quantitative analysis further confirmed that C5aR staining was significantly higher in pterygium tissues than in normal conjunctiva, with mean AOD values of 0.31 ± 0.04 and 0.13 ± 0.01, respectively (*P* < 0.001).

PCECs were successfully cultured from conjunctival explants. After 24 hours, cells were observed migrating out of the tissue blocks. To further verify whether the cultured cells were conjunctival epithelial cells, this study performed pan-keratin IF staining on the cultured cells. The results showed that these cells highly expressed pan-keratin protein, and the protein was mainly distributed in the cytoplasm (Fig. 6C), further confirming that the cultured cells were PCECs. Immunofluorescence in cultured PCECs further showed membrane-associated expression of C5aR (Fig. 6D), supporting their suitability for subsequent C5a stimulation experiments.

### Effects of C5a on the Biological Behavior of Primary Conjunctival Epithelial Cells

**Figure 7.**
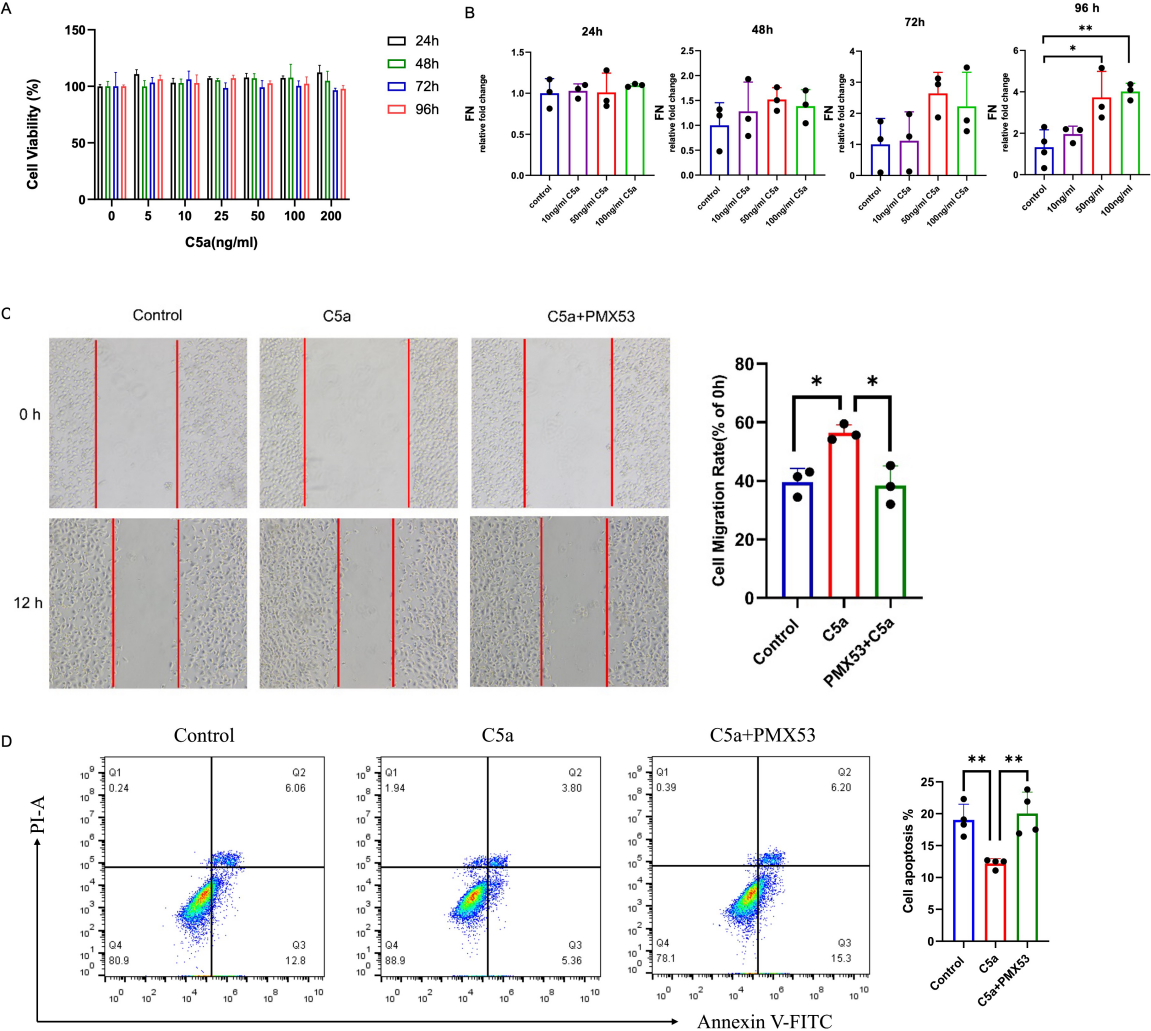
**Functional effects of C5a on conjunctival epithelial cells.** (**A**) CCK-8 proliferation assay: no significant proliferative changes were observed in PCECs treated with C5a (0–200 ng/mL) for 24 to 96 hours versus untreated controls (*n* = 3, *P* > 0.05). (**B**) Fibronectin (FN) mRNA expression: RT-qPCR analysis showing time- and concentration-dependent FN upregulation in C5a-treated cells. Significant induction occurred at 96 hours with 50 ng/mL C5a (*P < 0.05, **P < 0.01 vs. control; *n* = 3). (**C**) Scratch wound healing assay: C5a (50 ng/mL) significantly enhanced cell migration at 12 hours (*P < 0.05 vs. control), an effect blocked by co-treatment with C5aR antagonist PMX53 (50 nM). Migration rates quantified from three independent experiments. (**D**) Apoptosis analysis: Left panels show Annexin V-FITC/PI dot plots for the control, C5a-treated, and C5a+PMX53 co-treated groups (Q2+Q3 represent apoptotic cells); *right panel* shows statistical results, demonstrating significantly lower apoptosis in the C5a group than in the control, and restored apoptosis in the C5a+PMX53 group.

To investigate the biological effects of C5a on PCECs, we first assessed cell proliferation. CCK-8 analysis showed that C5a treatment (0–200 ng/mL) for 24 to 96 hours did not significantly affect cell proliferation compared with controls (Fig. 7A), indicating that C5a had no appreciable proliferative effect under these conditions.

Given that the expression of C5 in pterygium has been confirmed to be significantly higher than that in conjunctiva, and that conjunctival epithelial cells express C5aR, we further examined whether C5a stimulation was associated with EMT-related changes in PCECs. RT-qPCR revealed time-dependent FN upregulation: significant induction occurred at 96 hours (Fig. 7B), with 50 ng/mL identified as the minimal effective concentration. Morphological changes from a cobblestone-like to a spindle-shaped appearance further supported EMT-related phenotypic changes.

We next examined cell migration and apoptosis. The wound-healing assay demonstrated that 50 ng/mL C5a significantly accelerated wound closure after 12 hours (Fig. 7C). Co-treatment with the C5aR antagonist PMX53 (50 nM) markedly attenuated this promigratory effect, supporting the involvement of C5aR signaling.

Flow cytometric analysis further showed that C5a significantly reduced the apoptosis rate of PCECs compared with the control group (12.20% ± 0.74% vs. 19.03% ± 2.46%, *P* < 0.001), whereas co-treatment with PMX53 reversed this anti-apoptotic effect (20.03% ± 3.37% vs. 12.20% ± 0.74%, *P* < 0.05; Fig. 7D). These results suggest that C5a promotes migration and cell survival in conjunctival epithelial cells, at least partly through C5aR signaling.

### C5a Promotes EMT-Related Changes via C5aR Signaling

**Figure 8.**
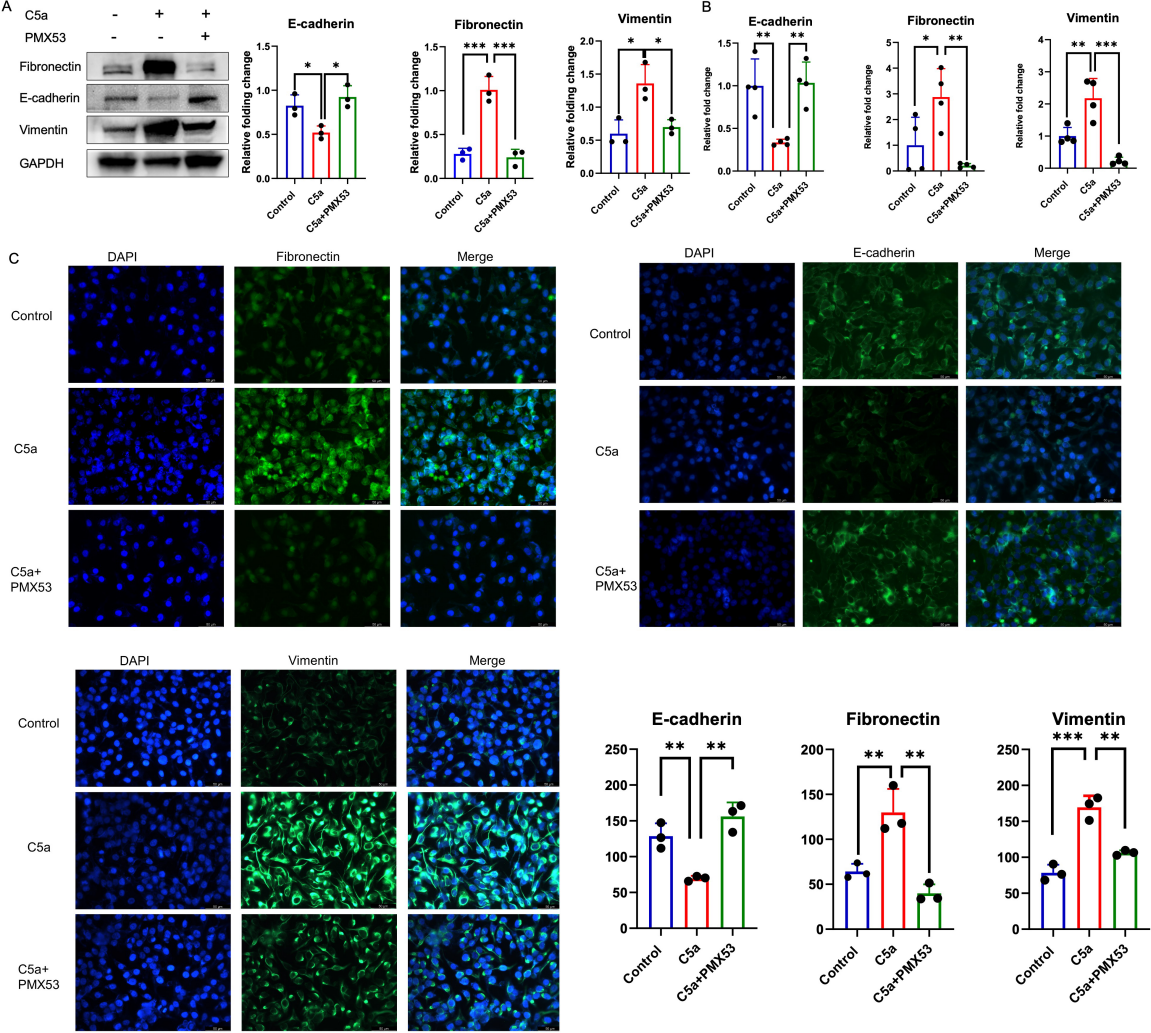
**C5a induces EMT through C5aR-dependent signaling in conjunctival epithelial cells.** (**A**) Western blot analysis: C5a significantly upregulated mesenchymal markers vimentin and FN while downregulating the epithelial marker E-cadherin; these changes were blocked by PMX53 co-treatment. *Right panels* show quantitative analysis of relative protein expression (*n* = 3). (**B**) RT-qPCR verification of EMT marker mRNA levels: consistent with WB results, RT-qPCR showed that C5a treatment significantly increased vimentin and FN mRNA expression and decreased E-cadherin mRNA expression; these effects were reversed by PMX53 co-treatment. Bar graphs show quantitative analysis of relative gene expression (*n* = 4). (**C**) Immunofluorescence staining of EMT markers for subcellular localization and expression: all immunofluorescence experiments used DAPI (*blue*) for nuclear staining and green fluorescence for target proteins; *right panels* show quantitative analysis of fluorescence (*n* = 3). *P < 0.05; **P < 0.01; ***P < 0.001, 1-way ANOVA followed by Tukey's multiple comparison test.

To further clarify the effect of C5a on conjunctival epithelial cells, we evaluated EMT-associated markers at both the protein and gene levels. At the protein level, WB results showed that after 96 hours of C5a treatment, the mesenchymal markers vimentin and FN were significantly upregulated, whereas the epithelial marker E-cadherin was significantly downregulated (*P* < 0.05; Fig. 8A). Cell immunofluorescence further showed changes consistent with EMT-related marker alterations (Fig. 8C). After 96 hours of C5a treatment, compared with the control group, the expression of mesenchymal markers (vimentin and FN) was upregulated, and the epithelial marker E-cadherin was significantly downregulated (*P* < 0.05). At the gene level, RT-qPCR also showed that compared with the control group, the expression of mesenchymal markers (vimentin and FN) was upregulated, whereas the expression of the epithelial marker E-cadherin was downregulated (Fig. 8B), with statistically significant differences (*P* < 0.05).

To determine whether C5a-associated EMT-related changes in conjunctival epithelial cells is mediated by its receptor C5aR, the specific C5aR antagonist PMX53 was used. As shown by WB and RT-qPCR (see Figs. 8A, 8B), co-treatment with PMX53 (50 nM) prevented C5a-induced upregulation of vimentin and FN, as well as the downregulation of E-cadherin. This was also confirmed by IF (see Fig. 8C). These results support the involvement of C5aR signaling in C5a-associated EMT-related changes in conjunctival epithelial cells.

### C5a Increases VEGFA Expression

H&E staining and proteomic analyses in this study indicated an increase in neovascularization and vascular remodeling in pterygium. As a key regulatory factor in angiogenesis, VEGFA plays a central role in vascular development and regeneration. Therefore, this study further investigated the expression changes of VEGFA in pterygium and the effect of C5a on its expression.

**Figure 9.**
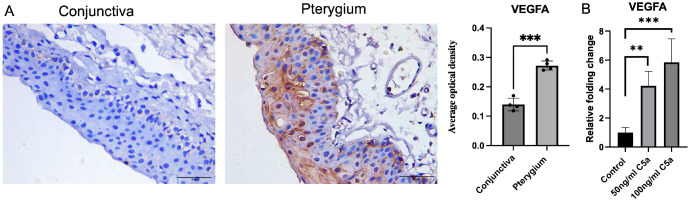
**C5a induces VEGFA expression in pterygium and conjunctival epithelial cells.** (**A**) VEGFA IHC in normal conjunctiva and pterygium, with semi-quantitative analysis by average optical density. *Scale bar* = 50 µm. (**B**) Bar graph depicting the relative fold change of VEGFA expression in conjunctival epithelial cells treated with 50 ng/mL or 100 ng/mL C5a for 96 hours, compared with untreated controls. **P < 0.01, ***P < 0.001 versus control group.

First, IHC for VEGFA in pterygium and normal conjunctival tissues revealed that the expression of VEGFA in pterygium was significantly higher than that in normal conjunctiva (Fig. 9A). Semi-quantitative analysis further confirmed that VEGFA staining was significantly increased in pterygium tissues compared with normal conjunctiva, with mean AOD values of 0.27 ± 0.02 and 0.14 ± 0.02, respectively (*P* < 0.001). In normal conjunctiva, VEGFA was mainly localized in the epithelial layer and vascular endothelial cells; within the epithelial layer, its expression was most concentrated in the basal layer, and this distribution was broadly consistent with the localization of C5aR expression. In pterygium tissues, VEGFA was also expressed in the epithelial layer and vascular endothelial cells. However, unlike its expression pattern in normal conjunctiva, VEGFA was diffusely distributed in the epithelial layer, with high expression also observed in the superficial cells.

To further understand the effect of C5a on conjunctival epithelial cells, we detected the expression of VEGFA in conjunctival epithelial cells after C5a treatment. As shown in Figure 9B, the production of VEGFA was influenced by C5a in a concentration-dependent manner. Treatment with 50 ng/mL C5a for 96 hours significantly increased VEGFA expression, and this increase was even more pronounced after treatment with 100 ng/mL C5a for 96 hours.

## Discussion

By integrating exploratory proteomic screening with tissue-level and functional validation, this study supports the involvement of C5a/C5aR signaling in EMT-related epithelial changes in pterygium. C5a may act through its receptor C5aR on the surface of conjunctival epithelial cells, promoting EMT-like changes and thereby contributing to fibrovascular remodeling in pterygium. These findings provide a new perspective on the molecular mechanisms underlying pterygium and may help inform future targeted therapeutic strategies.

Our proteomic analysis revealed changes consistent with an EMT signature in pterygium, including downregulation of E-cadherin and upregulation of FN, together with ECM-related proteins such as COL6A3 and MMP-2. Histopathological changes, particularly disrupted cell-cell junctions, further supported the presence of EMT. Previous studies also support the relevance of EMT in pterygium. Microarray analyses have revealed differential miRNA expression in pterygium, supporting the involvement of EMT-related regulation at the gene regulatory level.^19^^,^^20^ Zhang et al.^12^ demonstrated through single-cell RNA sequencing that EMT-related epithelial cells were significantly increased, highlighting the critical role of epithelial EMT in pterygium pathogenesis. Molecular assays have also consistently documented epithelial-mesenchymal marker shifts.^15^^,^^20^^,^^21^ Taken together, these findings support the view that conjunctival EMT represents an important pathogenic process in the fibrovascular invasion of pterygium and suggest that modulation of EMT may have therapeutic relevance.

C5 emerged as a candidate molecule potentially associated with pterygium pathogenesis. Proteomic screening revealed consistent upregulation of complement component C5 across both cohorts; KEGG pathway enrichment analysis identified the complement and coagulation cascade as the significantly enriched pathway for DEPs, aligning with transcriptomic findings by Bang et al.^22^ ELISA further confirmed increased expression of C5 and C5a in pterygium tissues. Notably, our clinical correlation analysis demonstrated a significant positive association between C5 expression levels and pterygium vascular density, suggesting a potential role of C5 in vascular-related pathological changes in pterygium. As a critical nexus bridging innate and adaptive immunity, the complement system contributes to pathological processes by regulating immune responses and inflammation. Within this cascade, C5 occupies a central position.^23^ Upon activation, C5 is cleaved into C5a and C5b fragments, with C5a serving as the major bioactive effector that amplifies inflammatory cascades through chemotactic recruitment of immune cells, such as neutrophils and monocytes.^24^ This is compatible with the extensive inflammatory cell infiltration and immunoglobulin deposition (IgG/IgA/IgM) observed in pterygium.^25^^–^^27^

Functionally, our results provide evidence that C5a promotes EMT-like changes in conjunctival epithelial cells. Upon C5a exposure, these cells exhibited features consistent with EMT: molecular analyses demonstrated upregulation of mesenchymal markers and downregulation of epithelial markers. In parallel, functional assays showed that C5a enhanced cellular migration and reduced apoptosis, both of which are consistent with EMT-related cellular behavior. Consistent with our findings, Salvador et al.^28^ demonstrated that C5a can similarly promote EMT-related changes in retinal pigment epithelial cells. Additionally, C5a has been shown to promote mesenchymal transition-related processes in macrophages,^29^ pericytes,^30^ epithelial cells,^31^ and endothelial cells.^32^ To determine whether C5a-induced EMT in conjunctival epithelial cells was mediated through C5aR, we performed co-treatment experiments using PMX53, a pharmacologic C5aR antagonist. PMX53 significantly attenuated the EMT changes induced by C5a, supporting the involvement of C5aR signaling in this process. Because PMX53 was used as a pharmacologic inhibitor, these findings should be interpreted as supportive evidence for the role of C5aR signaling rather than definitive proof of absolute target specificity. This aligns with previous studies demonstrating that deletion or blockade of C5aR effectively alleviates fibrosis in the renal tubulointerstitium,^33^ pancreas,^34^ glomeruli,^32^ lungs,^35^ and retina.^28^ Taken together, our findings support the view that C5a may promote EMT-like changes in conjunctival epithelial cells through C5aR-dependent signaling.

Beyond its association with EMT, C5a may also contribute to pterygium pathogenesis by promoting angiogenesis. Our data demonstrate that C5a upregulates VEGFA in conjunctival epithelial cells, which may have important pathological implications. IHC analyses revealed significantly increased VEGFA expression in pterygium epithelium compared with normal conjunctiva, with a broader epithelial distribution rather than confinement mainly to the basal layer. As a key driver of angiogenesis, VEGF promotes endothelial cell migration and neovascularization, thereby potentially contributing to pterygium progression.^36^ This mechanistic link is supported by clinical evidence: anti-VEGF therapies have shown efficacy as adjuvants in pterygium surgery,^37^ supporting the biological relevance of VEGFA as a potential therapeutic target. Notably, our findings align with broader evidence of C5a-mediated VEGF regulation: C5a has been shown to induce VEGF secretion in human retinal pigment epithelial cells,^38^ while Kurihara et al.^39^ demonstrated that C5a directly promotes endothelial cell migration, proliferation, and vasculogenesis.

Based on these findings, we propose that external stimuli, such as ultraviolet radiation or microbial infection, may activate the local complement system, leading to C5 cleavage and C5a generation. C5a may then act on conjunctival epithelial cells through C5aR, promoting EMT-like changes and increasing VEGFA expression. Together, these processes may facilitate fibrovascular growth and contribute to the characteristic pathological phenotype of pterygium. To our knowledge, this study is among the first to link complement activation with EMT-related changes in pterygium and to implicate the C5a/C5aR axis in this process. Given that complement-targeted therapies such as C5aR antagonists have already entered clinical use in other diseases,^40^ our findings support the potential relevance of complement signaling as a therapeutic target in pterygium.

Despite providing important insights into pterygium, this study has several limitations. First, the control conjunctiva was obtained from relatively normal conjunctiva of pterygium patients and may therefore contain subtle pre-pathological molecular changes. Second, the initial proteomic screening was based on nominal *P* values rather than false discovery rate (FDR)-adjusted thresholds; thus, the DEP-level findings should be interpreted as exploratory and hypothesis-generating rather than definitive biomarker evidence. However, the main conclusions were supported by cross-cohort reproducibility and orthogonal validation. Moreover, because the correlation and ROC analyses were performed in the discovery cohort, their clinical relevance and diagnostic utility require confirmation in larger independent cohorts. Third, although our findings support the involvement of C5a/C5aR signaling, downstream mechanisms were not fully delineated. In addition, PCECs are useful for modeling early epithelial responses and pathogenic induction but do not fully recapitulate the complex microenvironment of established pterygium tissue, and the lack of a well-established animal model precluded in vivo validation. Future studies should include healthy conjunctival controls, larger independent cohorts, pterygium-derived cell models, and complementary in vivo approaches.

## Conclusions

This study supports the potential involvement of C5a/C5aR signaling in EMT-related epithelial changes in pterygium. These findings provide additional insight into pterygium pathogenesis and suggest that C5aR may represent a potential therapeutic target for future investigation.

## Supplementary Material

Supplement 1
