# Supplementary material for: Complement C5a Promotes Epithelial-Mesenchymal Transition in Pterygium via C5aR Activation
Source: Invest Ophthalmol Vis Sci. 2026 Jun 10;67(6):16. doi: 10.1167/iovs.67.6.16 (PMC13263985; doi:10.1167/iovs.67.6.16)
Supplement: Supplement 1 [file iovs-67-6-16_s001.pdf]

Supplementary File S1. Detailed LC-MS/MS Parameters for Proteomic Analysis

Parameter	Discovery Cohort (Label-Free)	Validation Cohort (4D Label-Free)
LC System	EASY-nLC 1200	EASY-nLC 1200
Trap Column	C18 (3 µm, 75 µm × 2 cm)	C18 (3 µm, 100 µm × 2 cm)
Analytical Column:	C18 (2 µm, 75 µm × 25 cm)	C18 (1.9 µm, 150 µm ×30 cm)
LC Mobile Phase A	0.1% formic acid in water	0.1% formic acid in water
LC Mobile Phase B	0.1% formic acid in 80% acetonitrile	0.1% formic acid in 80% acetonitrile
LC Gradient (Time-% B)	0–2 min, 3%–8% B; 2–12 min, 8%–10% B; 12–22 min, 10%–15% B; 22–37 min, 15-25% B; 37–47 min, 25-32% B; 47–57 min, 32-100% B; and 57–67 min,100% B.	0min, 4% B; 0-10 min, 4%-15% B; 10-125 min, 15%-30% B; 125-140 min, 30%-50% B; 140-141 min, 50%-100% B; 141-150 min, 100% B
LC Flow Rate	300 nL/min	600 nL/min
MS	Orbitrap Fusion Lumos Tribrid	Orbitrap Exploris 480 with FAIMS
MS1 Resolution	60,000	120,000
MS1 Scan Range (m/z)	350 - 1500	300 - 1400
Automatic gain control	5E4	5E4
MS2 Analysis	Orbitrap (Resolution: 15,000)	Ion Trap (Rapid mode)
FAIMS CV Values	N/A	-45 V, -65 V

LC=liquid chromatography, CV=compensation voltages, MS= mass spectrometer.

Supplementary File S2. Diagnostic Value of the 23 Upregulated DEPs

DEP	AUC	95% CI
COL6A3	0.86	0.696 - 1.000
HBA1; HBA2	0.82	0.632 - 1.000
SPTA1	0.86	0.691 - 1.000
COL12A1	0.85	0.678 - 1.000
HBB	0.84	0.664 - 1.000
CAT	0.79	0.580 - 1.000
BLVRB	0.9	0.766 - 1.000
SLC4A1	0.87	0.710 - 1.000
CA1	0.84	0.664 - 1.000
C5	0.8	0.602 - 0.998
CA2	0.81	0.610 - 1.000
TPM1	0.95	0.865 - 1.000
RPIA	0.96	0.875 - 1.000
PLA2G2A	0.96	0.875 - 1.000
SUSD2	0.8	0.593 - 1.000
HBE1	0.9	0.766 - 1.000
HAGH	0.87	0.697 - 1.000
KANK2	0.88	0.727 - 1.000
ADD2	0.92	0.801 - 1.000
DMTN	0.9	0.754 - 1.000
PLPP3	0.8	0.600 - 1.000
THBS1	0.82	0.618 - 1.000
F9	0.86	0.686 - 1.000

DEP=differentially expressed protein, AUC= area under the curve, CI=confidence interval.
